# Passive Millimeter Wave Imaging System Based on Helical Scanning

**DOI:** 10.1038/s41598-018-25637-9

**Published:** 2018-05-18

**Authors:** Yang Meng, Anyong Qing, Chuan Lin, Jiefeng Zang, Yizhe Zhao, Cheng Zhang

**Affiliations:** 1 University of Electronic Science and Technology of China, Chengdu, 610054 China; 2 Southwest Jiaotong University, Chengdu, 610031 China

## Abstract

A simple and fast single channel passive millimeter wave (PMMW) imaging system for public security check is presented in this paper. It distinguishes itself with traditional ones by an innovative scanning mechanism. Indoor experiments against human body with or without concealed items in clothes show that imaging could be completed in 3 s with angular resolution of about 0.7°. In addition, its field of view (FOV) is adjustable according to the size of actual target.

## Introduction

Millimeter wave (MMW) has emerged as a promising innovative approach for security check due to its penetration capability and high resolution. More importantly, according to the principle of blackbody radiation, each and every object with temperature above absolute zero emits characteristic MMW carrying intrinsic information about itself. The object could be non-invasively identified by receiving the autonomous MMW radiation from the object only. In principle, there is no artificial MMW source in a PMMW imaging system to illuminate objects of interest. Hence, it is absolutely safe to both inspectors and inspected objects. It is one of the critical features taken advantage of by PMMW imaging technology for diverse promising applications^1,2^, e.g., security check at airports, railway and subway stations, *etc*^3^. PMMW imaging technology has been being a hot research and engineering topic in recent years^4–12^.

Generally speaking, at present, there are four types of PMMW imaging systems: mechanical scanning, phased-array, synthetic aperture and focal-plane array (FPA), among which the single channel mechanical scanning imaging system is currently more favorable due to its simplicity and low cost.

Millivision^13^ developed one of the earliest single channel mechanical scanning imaging systems. It takes about 30 minutes to obtain an image. QinetiQ’s system^14^ implemented focusing antennas and conical scanning. It is faster but its structure is too complex. The opto-mechanical scanning system developed by Beijing Institute of Technology^15,16^ employed a crank-rocker mechanism to generate high speed line scanning. The imaging time is longer than 20 seconds. Moreover, the field of view is limited.

In this paper, a simple and fast single channel system is presented. It distinguishes itself with traditional ones by an innovative scanning mechanism, namely the helical scanning. Indoor experiments against human body with or without concealed items in clothes confirm its desirable performance.

## Fundamental Theory

### Radiation of Millimeter Waves

It is well known that all natural objects with absolute temperature above zero emit electromagnetic radiation including millimeter wave. According to Planck’s law, a blackbody radiates uniformly in all directions with a spectral energy density *u* given by^17^

1 $$u(f,T)=\frac{8\pi h{f}^{3}}{{c}^{3}}(\frac{1}{{e}^{hf/kT}-1})$$

where *f* is frequency, *T* is the absolute temperature of the object in K, *h* is Planck’s constant (6.62606896 × 10^−34^ J·s)*, k* is Boltzmann constant (1.38054 × 10^−23^ J·K^−1^), *c* is the speed of light (2.9979 × 10^8^ m·s^−1^).

In the limit of low frequencies, Planck’s law tends to the Rayleigh–Jeans approximation

2 $$u(f,T)=\frac{8\pi k{f}^{2}T}{{c}^{3}}$$

Within the band centered at frequency *f* with bandwidth Δ*f*, the emission energy from unit volume of an object is given by

3 $$E(f,T)=\rho u{\rm{\Delta }}f=\rho \frac{8\pi k{f}^{2}T{\rm{\Delta }}f}{{c}^{3}}$$

where 0 ≤ *ρ* ≤ 1 is the emissivity of the object.

### Effective Radiation Temperature

Besides autonomous radiation of MMW, an object also reflects and transmits MMW incident on it. Ultimately, the object can be represented by an effective radiation temperature *T*_*E*_^18,19^

4 $${T}_{E}=\rho {T}_{0}+r{T}_{I}+t{T}_{B}$$

where *r* and *t* are reflectivity and transmittance of the object, *T*_*I*_ is the absolute temperature of sources in front of it that it reflects, and *T*_*B*_ is the absolute temperature of sources behind it that it transmits, *ρ* + *r* + *t* = 1 according to the law of energy conservation. Values of *r*, *t*, and *ρ* of some representative materials in security check are given in Table 1.

**Table 1:** Properties of Representative Materials in W Band.

Material	*r*	*t*	*ρ*
Skin	0.2	0	0.8
Cloth	0.01	0.91	0.08
Metal	1	0	0
Plastics	0.26	0	0.74
Ceramics (zro_2_)	4/9	1/9	4/9

The emissivity *ρ*, reflectivity *r*, and transmittance *t*, of a material is uniquely determined by its constitutive parameters, incident angle, and polarization of incident waves. According to electromagnetic theory, at normal incidence, we have

5 $$r={|\frac{Z-{Z}_{0}}{Z+{Z}_{0}}|}^{2}$$

6 $$t={|\frac{2Z}{Z+{Z}_{0}}|}^{2}$$

where $Z=\sqrt{\mu /\varepsilon }$ is the intrinsic impedance of the object, *μ* is permeability and *ε* is permittivity, ${Z}_{0}=\sqrt{{\mu }_{0}/{\varepsilon }_{0}}$ is the intrinsic impedance of free space, *μ*_0_ is the permeability of free space and *ε*_0_ is the permittivity of free space.

### Passive Millimeter Wave Imaging

Passive millimeter wave (PMMW) imaging systems measure the distribution of effective radiation temperatures of concerned objects. In laboratory experiment, the received MMW presents the integrated effect of the tested objects of interest, volunteer’s body, clothes, and the test environment.

For the case of a clear volunteer, we have

7 $${T}_{Ec}={\rho }_{c}{T}_{c}+{r}_{c}{T}_{s}+{t}_{c}{T}_{Ev}$$

8 $${T}_{Ev}={\rho }_{v}{T}_{v}+{r}_{v}{T}_{Ec}+{t}_{v}{T}_{s}$$

where *T*_*Ec*_ is the effective radiation temperature in front of clothes, *ρ*_*c*_, *r*_*c*_, *t*_*c*_, and *T*_*c*_ are the emissivity, reflectivity, transmittance and absolute temperature of clothes, *T*_*s*_ is the absolute temperature of free space, *T*_*Ev*_ is the effective radiation temperature in front of the skin of the clear volunteer, *ρ*_*v*_, *r*_*v*_, *t*_*v*_, and *T*_*v*_ are the emissivity, reflectivity, transmittance and absolute temperature of the clear volunteer.

Therefore, the output of the PMMW imaging system reads

9 $$V\propto {T}_{Ec}=\frac{{\rho }_{c}{T}_{c}+{r}_{c}{T}_{s}+{t}_{c}({\rho }_{v}{T}_{v}+{t}_{v}{T}_{s})}{1-{t}_{c}{r}_{v}}$$

From now on, we will treat the output *V* of the PMMW imaging system and the effective radiation temperature *T*_*Ec*_ of the volunteer’s clothes interchangeably identical.

When suspicious objects are hidden in the volunteer’s clothes, we have

10 $${T}_{Ec}={\rho }_{c}{T}_{c}+{r}_{c}{T}_{s}+{t}_{c}{T}_{Eo}$$

11 $${T}_{Eo}={\rho }_{o}{T}_{o}+{r}_{o}{T}_{Ec}+{t}_{o}{T}_{Ev}$$

12 $${T}_{Ev}={\rho }_{v}{T}_{v}+{r}_{v}{T}_{Eo}+{t}_{v}{T}_{s}$$

where *T*_*Eo*_ is the effective radiation temperature in front of the object of interest, *ρ*_*o*_, *r*_*o*_, *t*_*o*_, and *T*_*o*_ are the emissivity, reflectivity, transmittance and absolute temperature of the object.

In accordance, the output of the PMMW imaging system is

13 $${T}_{Ec}=\frac{({\rho }_{c}{T}_{c}+{r}_{c}{T}_{s})(1-{t}_{o}{r}_{v})+{t}_{c}[{\rho }_{o}{T}_{o}+{t}_{o}({\rho }_{v}{T}_{v}+{t}_{v}{T}_{s})]}{1-{t}_{o}{r}_{v}+{t}_{c}{r}_{o}}$$

The effective radiation temperatures of the materials listed in Table 1 at different environment temperatures have been computed as shown in Table 2. It is observed that

1. For each and every material, the effective radiation temperature increases simultaneously with environment temperature.
2. The difference between the effective radiation temperature of clear volunteer and that of object of interest drops to nothing as the environment temperature approaches the volunteer’s body temperature. A moderately cool environment, e.g., 0 °C, is definitely more favorable.
3. Among all objects of interest, the difference between effective radiation temperature of the clear volunteer and that of metal object is always the largest. Therefore, metal object is the easiest object to detect by PMMW imaging system. This provides fundamental support to the test results presented shortly later.
4. The effective radiation temperatures of ceramics and plastics differ very little. Therefore, it is hardly possible for PMMW imaging systems to distinguish plastics from ceramics without extra information.

**Table 2:** Effective Radiation Temperatures of Different Materials.

Objects	*T*_*E*_			
	*T*_*s*_ = 0 °C	*T*_*s*_ = 20 °C	*T*_*s*_ = 37 °C	*T*_*s*_ = 40 °C
Clear volunteer	305.929	308.130	310.000	310.330
Metal	273.000	293.000	310.000	313.000
Plastics	289.057	300.377	310.000	311.698
Ceramics	291.271	301.395	310.000	311.519

## Prototype System

### System Design

**Figure 1:**
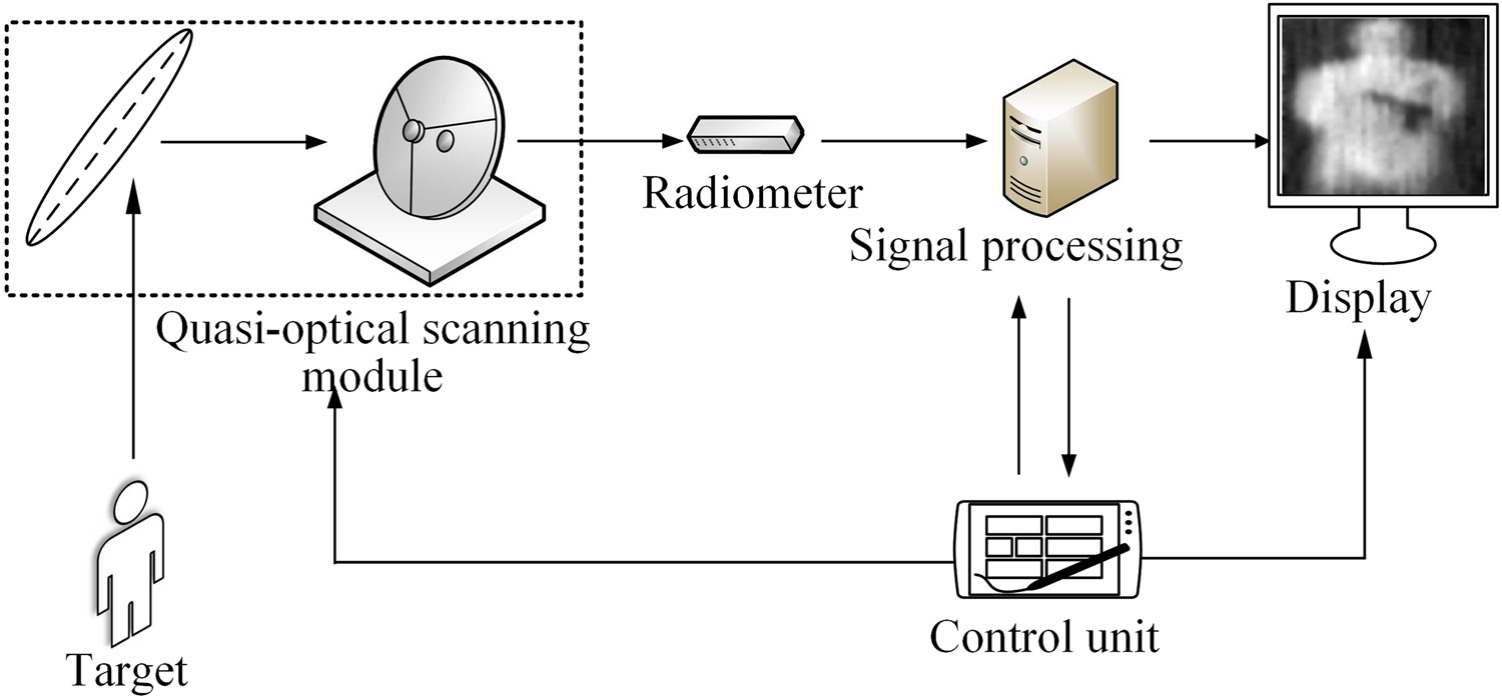
System Configuration of Prototyped PMMW Imaging System.

**Figure 2:**
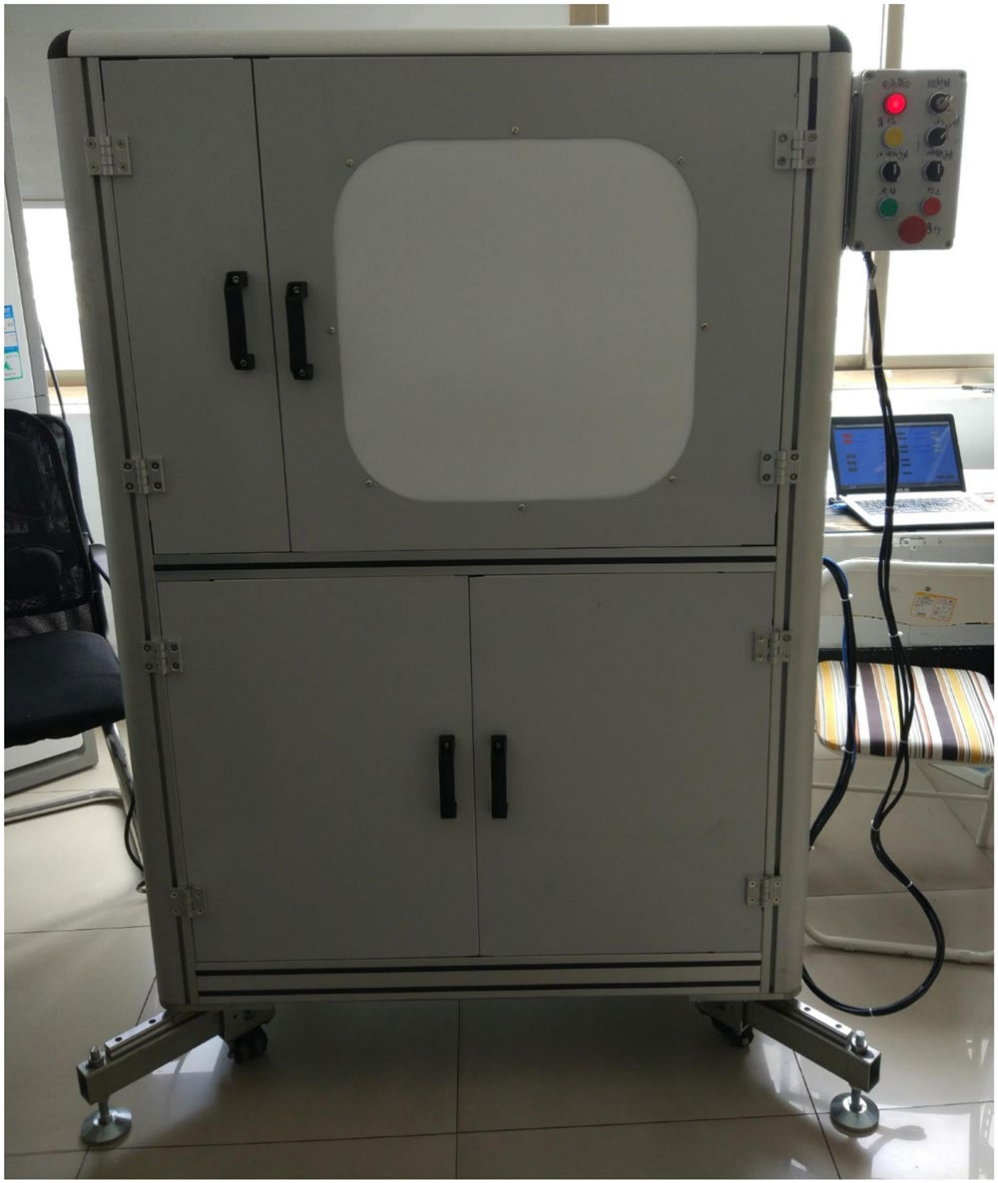
Prototyped PMMW Imaging System.

Our system works at the center frequency of 94 GHz. It is composed of quasi-optical scanning module, scanning control unit, W band radiometer, signal processing unit and display terminal as shown in Fig. 1. A photo of the prototyped PMMW system is given in Fig. 2. The upper layer is the quasi-optical scanning module and the lower layer stations the scanning control unit.

**Figure 3:**
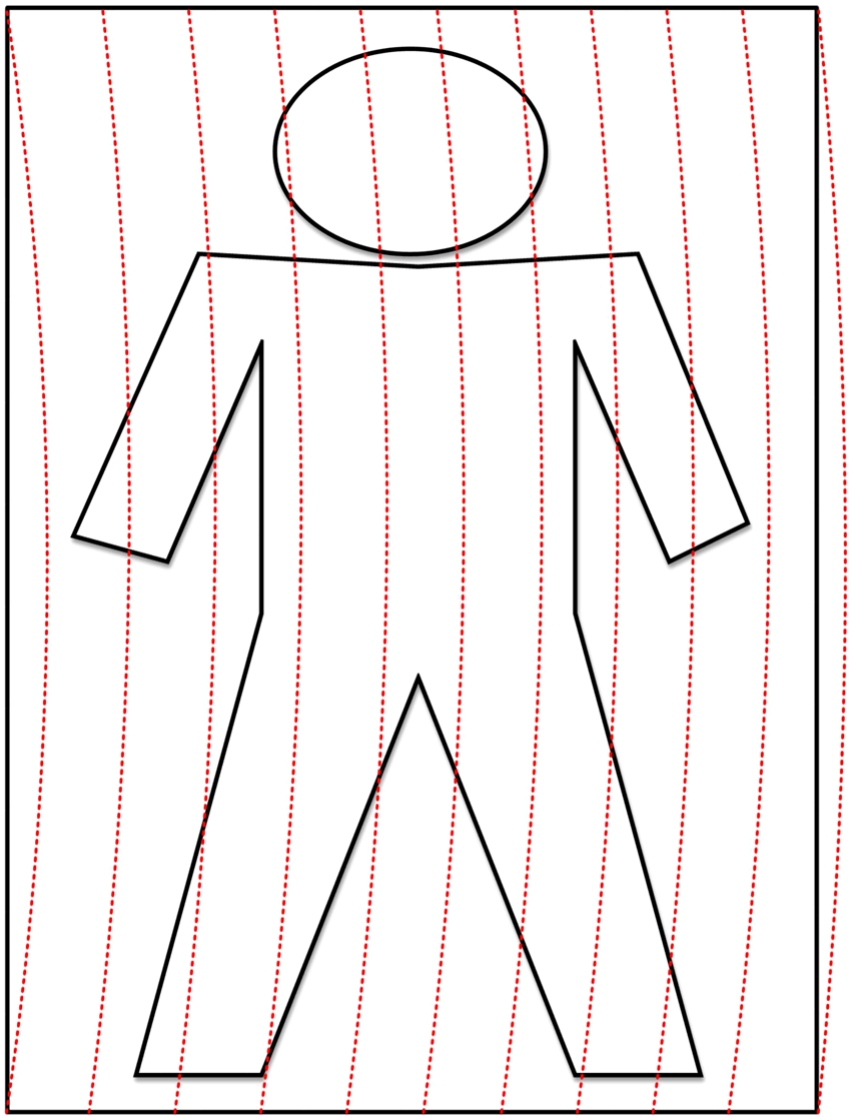
Trajectory of Helical Scanning.

With the help of two orthogonal turntables, a helical trajectory on the object as depicted in Fig. 3 is scanned. Detailed description about the helical trajectory will be described shortly. Millimeter waves from the target is reflected by the circular smooth metal reflector to the focusing antenna and focused in the feed. A Cassegrain antenna with 300 mm aperture size is selected as the focusing antenna. The focused signal is transmitted to the W band millimeter wave radiometer at the back of the focusing antenna through wave guide. Comprehensive evaluation of system performance, budget, and system complexity results in selection of the radiometer PMMW-10-0001 manufactured by Farran Technology Ltd. The radiometer consists of a millimeter wave low noise amplifier (LNA) cascade unit, a detector unit, and a video amplifier unit. Its Sensitivity @+25 °C is less than 4 K. Sampled voltage output from the radiometer proportional to the concerned scene radiation is then processed by in-house developed imaging algorithm. The distribution of effective radiation temperature is displayed on the terminal. It looks more or less like an optical image.

### Quasi-Optical Scanning System

**Figure 4:**
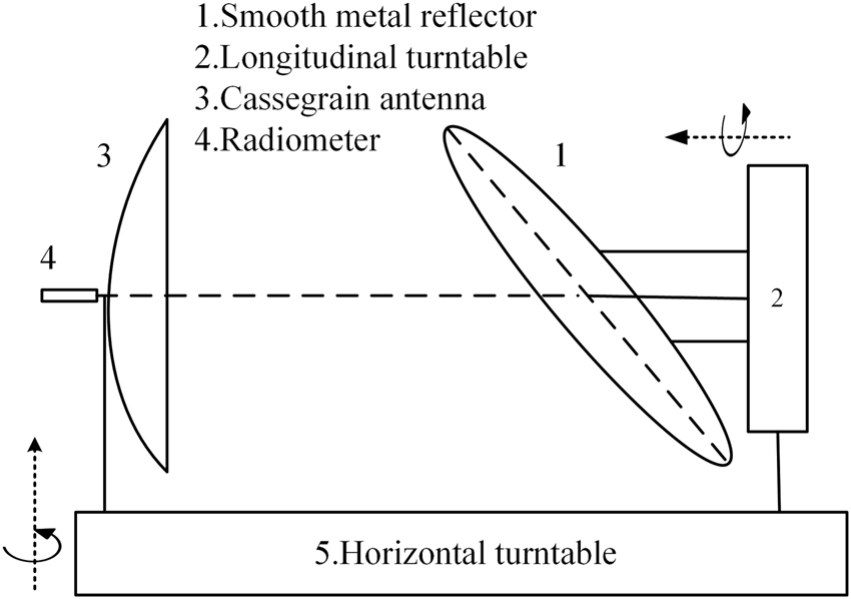
Quasi-optical Scanning System.

**Figure 5:**
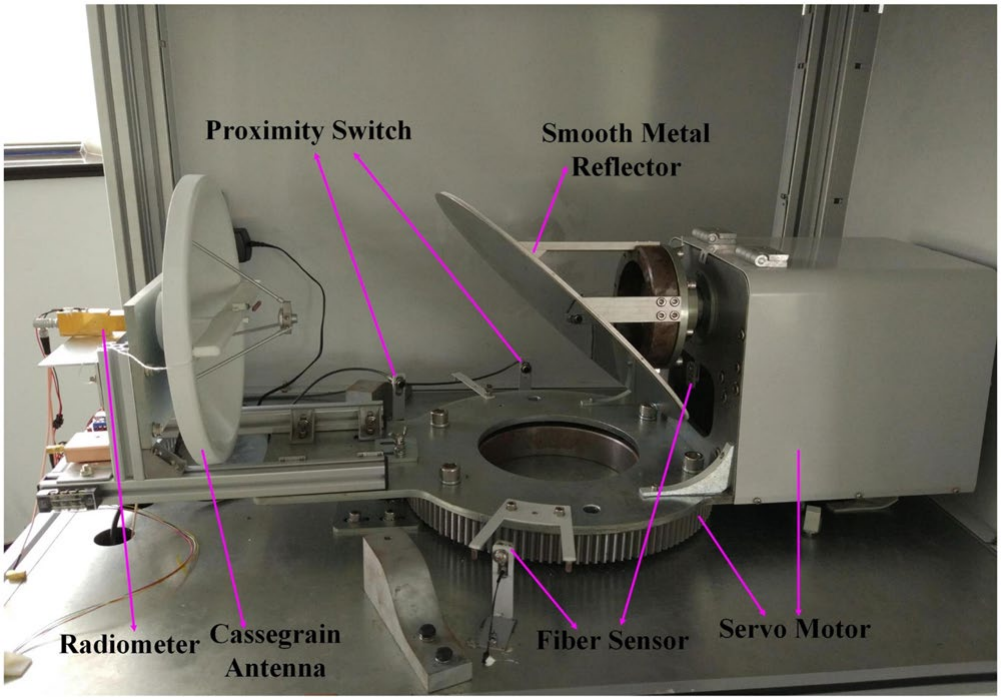
Photo of the Quasi-optical Scanning System in the Prototype System.

A novel quasi-optical scanning system as shown in Fig. 4 is invented to scan the object quickly. A photo of the quasi-optical scanning system in the prototype system is shown in Fig. 5. It consists of a Cassegrain focusing antenna, a circular smooth metal reflector, a longitudinal turntable, and a horizontal turntable.

As can be seen in Figs 4 and 5, the longitudinal turntable and the Cassegrain focusing antenna are coaxially mounted on opposite sides of the horizontal turntable while the circular smooth metal reflector is mounted on the longitudinal turntable. The normal of the reflector is 45° from the rotation axis of the longitudinal turntable. Obviously, one of the advantages of the novel scanning system points to its compactness.

The horizontal turntable swings around its vertical axis. Each one-way rotation corresponds to a single scan that produces an image of effective radiation temperature of the scanned object. Rotation is reversed between neighboring scans for faster scanning since it is unnecessary to reset the horizontal table.

The longitudinal turntable rotates around its horizontal axis. Unlike traditional translational scanning which periodically accelerates and decelerates, the longitudinal turntable rotates consistently, continuously and uniformly throughout normal operation of the PMMW imaging system. As such, the PMMW imaging system is mechanically more stable, and the scanning process is much smoother that the received signal is more reliable. It is believed to be one of the critical factors behind the success of the manufactured prototype.

### Data Acquisition

One fiber sensor is mounted on the base of the scanning module and two fiber covers are attached to the horizontal turntable to flexibly adjust the horizontal field of view on demand. In each scan, two pulses are generated in turn due to light blocking by the fiber covers. The first pulse triggers data acquisition and the second one stops data acquisition.

In each period of the longitudinal turntable, a column of data corresponding to a helical section of the object as shown in Fig. 3 is acquired. Similarly, acquisition of each data column in the scene is controlled by another fiber sensor and the other two fiber covers. The fiber sensor and fiber covers are carefully positioned so that the normal of the metal reflector reaches the target scene during the two pulse signals generated due to light blocking by the fiber covers. The data acquisition unit samples the output voltage signal of the radiometer when the first pulse signal is triggered, pause data acquisition when the second pulse signal is detected, and stays idle until awakened by the next pulse signal.

The core of the data acquisition unit is the AD7606 chip by Analog Device Inc. It is a 16-bit, simultaneous sampling, analog-to-digital data acquisition systems (DAS) with eight channels and 200 ksps on each channel.

**Figure 6:**
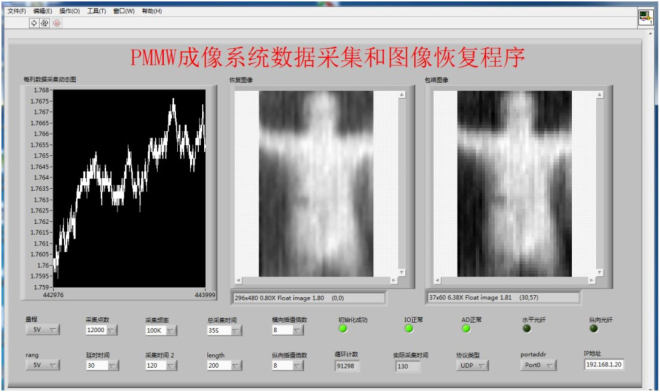
GUI of Prototype system.

A software to operate the data acquisition unit is developed in-house. All control parameters to operate the data acquisition unit are set through a user-friendly GUI as shown in Fig. 6. The GUI is developed by using LabVIEW Vision Development Module.

### Image Reconstruction

The acquired data is processed by in-house developed imaging software based on LabVIEW MathScript RT Module. The sampled voltage is first re-ordered according to the location information by making use of the pulse signals from the fiber sensors. Wavelet-based de-noising is then performed for higher signal-to-noise ratio. Finally, the processed data proportional to the concerned scene radiation will be visualized on the display terminal. Technical details of the reconstruction algorithm are available upon request through mail or email. A screenshot of the display terminal is given in Fig. 6.

### Performance Indicators

#### Efficiency

**Figure 7:**
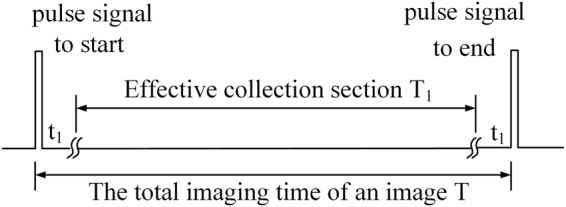
A Period of Horizontal Scan.

The period of each horizontal scan is *T*. As shown in Fig. 7, in each period, data acquisition starts at *t*_1_, lasts *T*_1_, and stops at *T* − *t*_1_. Therefore, the horizontal efficiency is

14 $${e}_{h}=\frac{{T}_{1}}{T}=1-\frac{2{t}_{1}}{T}$$

**Figure 8:**
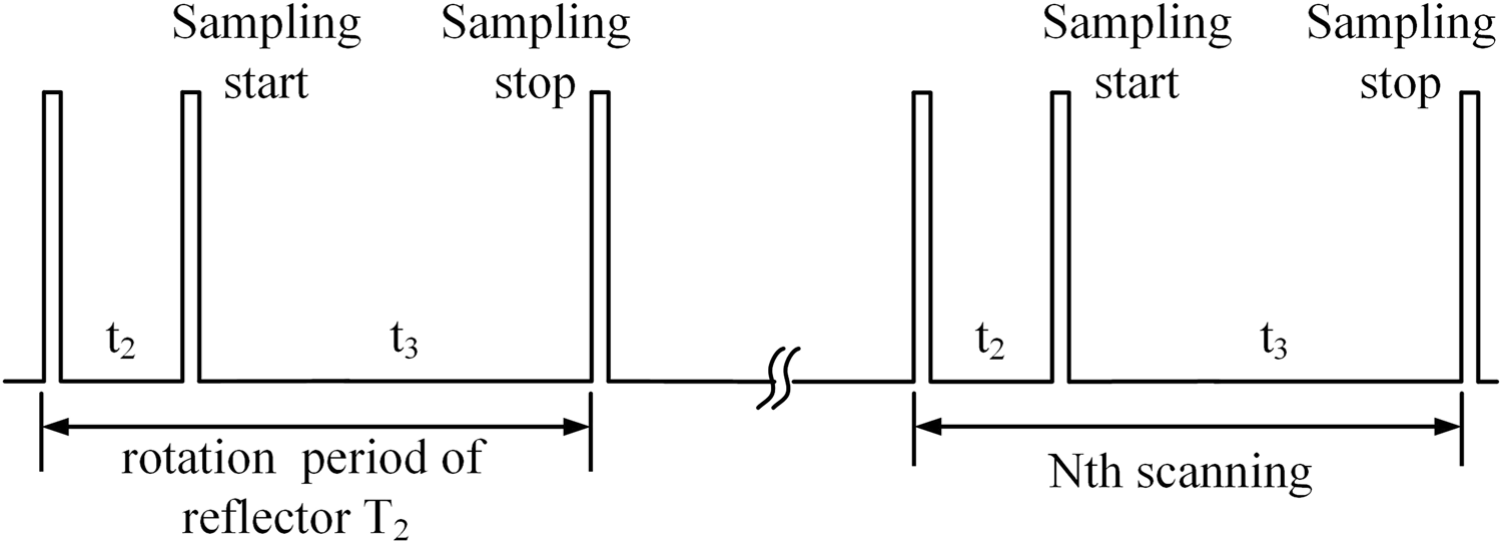
The Process of Data Acquisition.

As shown in Fig. 8, in each longitudinal period *T*_2_, data acquisition starts at *t*_2_ and lasts *t*_3_. Therefore, the longitudinal efficiency is

15 $${e}_{l}=\frac{{t}_{3}}{{T}_{2}}$$

In particular, as also shown in Fig. 8, we have

16 $${T}_{1}=N{T}_{2}$$

Ultimately, the efficiency of the novel PMMW imaging system is

17 $$e={e}_{h}{e}_{l}=\frac{{T}_{1}}{T}\frac{{t}_{3}}{{T}_{2}}=\frac{N{t}_{3}}{T}$$

For all the experiments presented in this paper, *T* ≈ 3 s, *T*_1_ = 2.7 s, *T*_2_ = 0.09 s, *N* = 30, *t*_3_ = 0.04 s. Therefore, the scanning efficiency of the prototype PMMW imaging system is about 40%.

#### Resolution

MMW radars usually employ narrow beam antennas for better angular resolution. In this regard, the angular resolution of MMW radars is generally represented by half power beam width *θ*_*h*_ of the antenna

18 $${\theta }_{h}={K}_{h}\frac{\lambda }{D}$$

where constant *K*_*h*_ is antenna-dependent, λ is the wavelength, and *D* is the aperture of the antenna. Empirically, ${K}_{h}=4/\pi$.

If the object stays at a distance *d* from the PMMW imaging system, the spatial resolution is

19 $${\rm{\Psi }}={\theta }_{h}d$$

Our system works at the center frequency of 94 GHz. A Cassegrain antenna with 300 mm aperture size is selected. According to Eq. (18), we have *θ*_*h*_ = 0.0127 (about 0.7°). Furthermore, If the distance between the antenna and target is 3 m, according to Eq. (19), the spatial resolution of the prototype system is about 38 mm which is confirmed by our indoor tests.

#### Fields of View

In principle, the horizontal field of view could be 360°. However, in practice, only objects in front of the PMMW imaging system is of interest. The field of view could be flexibly adjusted by the fiber sensor and fiber covers limit according to scenario of practical applications.

**Figure 9:**
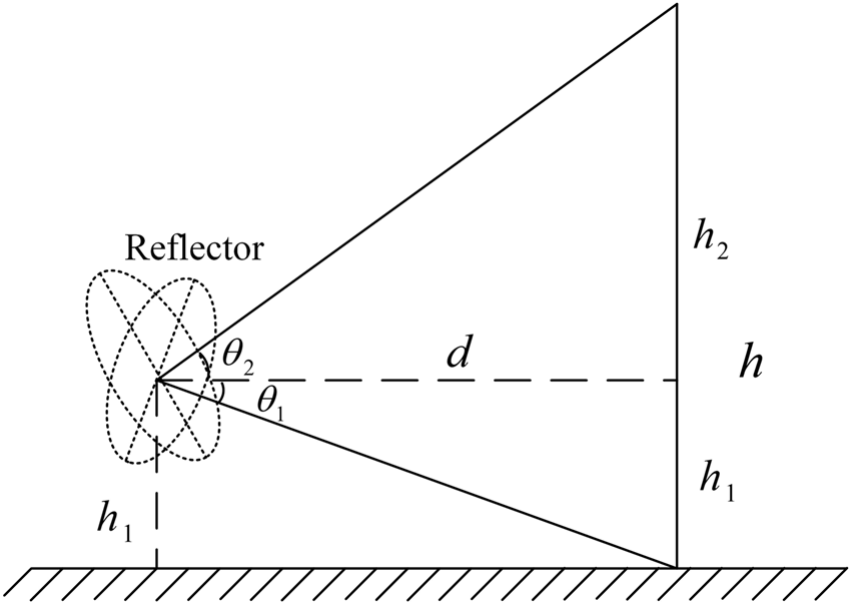
Field of view in longitudinal direction.

Similarly, there is no fundamental limit on the vertical field of view above the horizontal axis of the longitudinal turntable although a similar design is implemented to flexibly adjust the vertical field of view facing the object of interest. However, the vertical field of view is subject to the limit imposed by the height of the horizontal axis of the longitudinal turntable above ground, as shown in Fig. 9.

## Experiment Results

As mentioned before, the system is radiation free that no approval is necessary from any licensing committee. However, informed consent was obtained from all involved volunteers. All experiments were carried out in accordance with relevant guidelines and regulations.

### Clear Human Body

**Figure 10:**
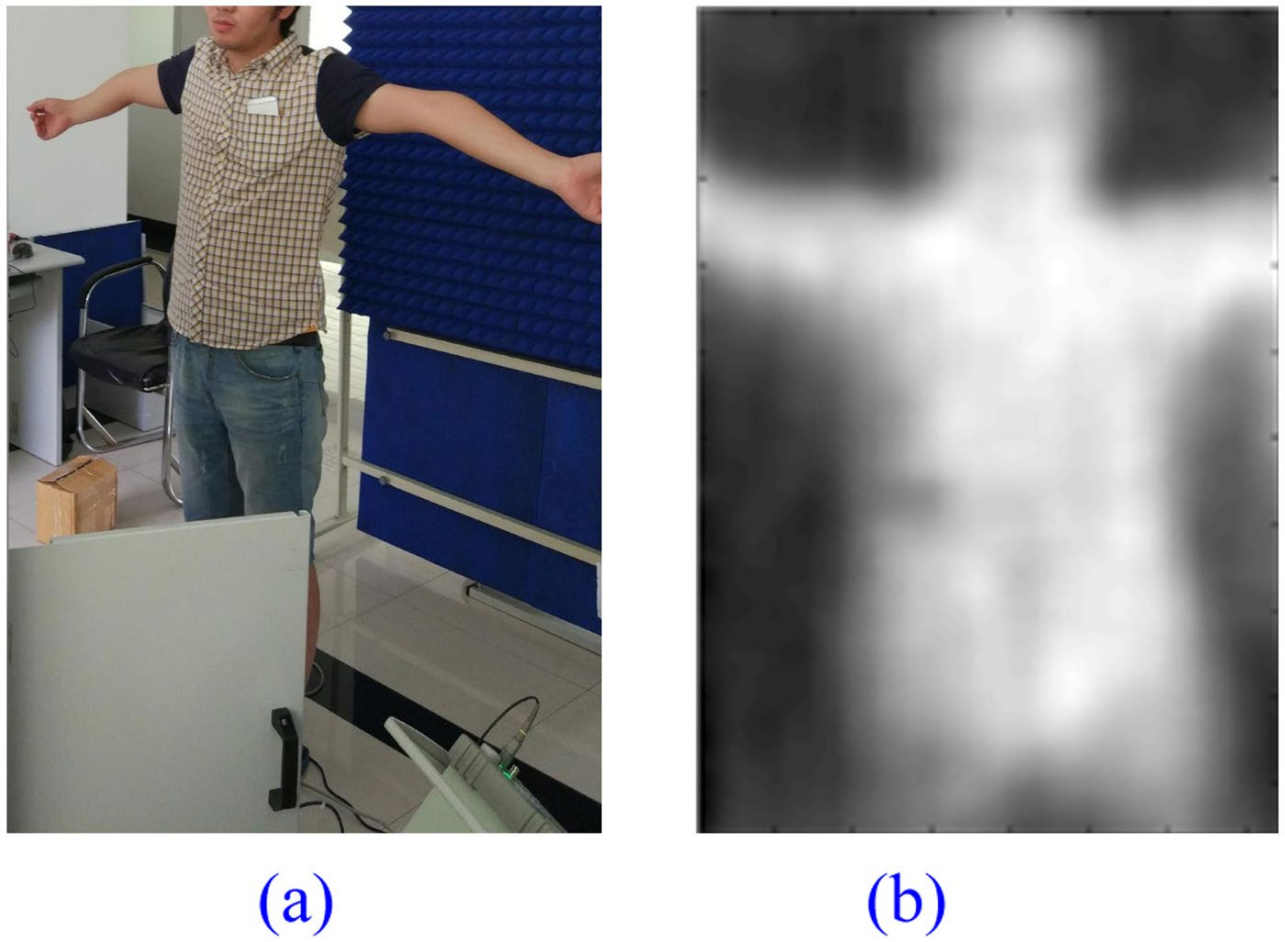
A Clear Volunteer. (**a**) Imaging Scene, (**b**)PMMW Image.

The prototype imaging system is first tested against clear human body without any concealed dangerous items in summer 2016. A photo of the indoor test scenario is shown in Fig. 10(a) while the image displayed on the system terminal is presented in Fig. 10(b). The volunteer under test stands 3 meters in front of the prototype system.

The temperature of normal human body keeps at about 37 °C while the room temperature in the workshop in summer is usually above 30 °C. As can be seen in Table 2, the difference between the effective radiation temperature of the clear volunteer and the environment temperature is relatively small. A microwave absorbing wall is therefore placed behind the volunteer to reduce the effect of obviously unfavorable test condition. Even under such challenging situation, the body outline and posture can be clearly identified in the image.

### I-Shape Metal Plate

**Figure 11:**
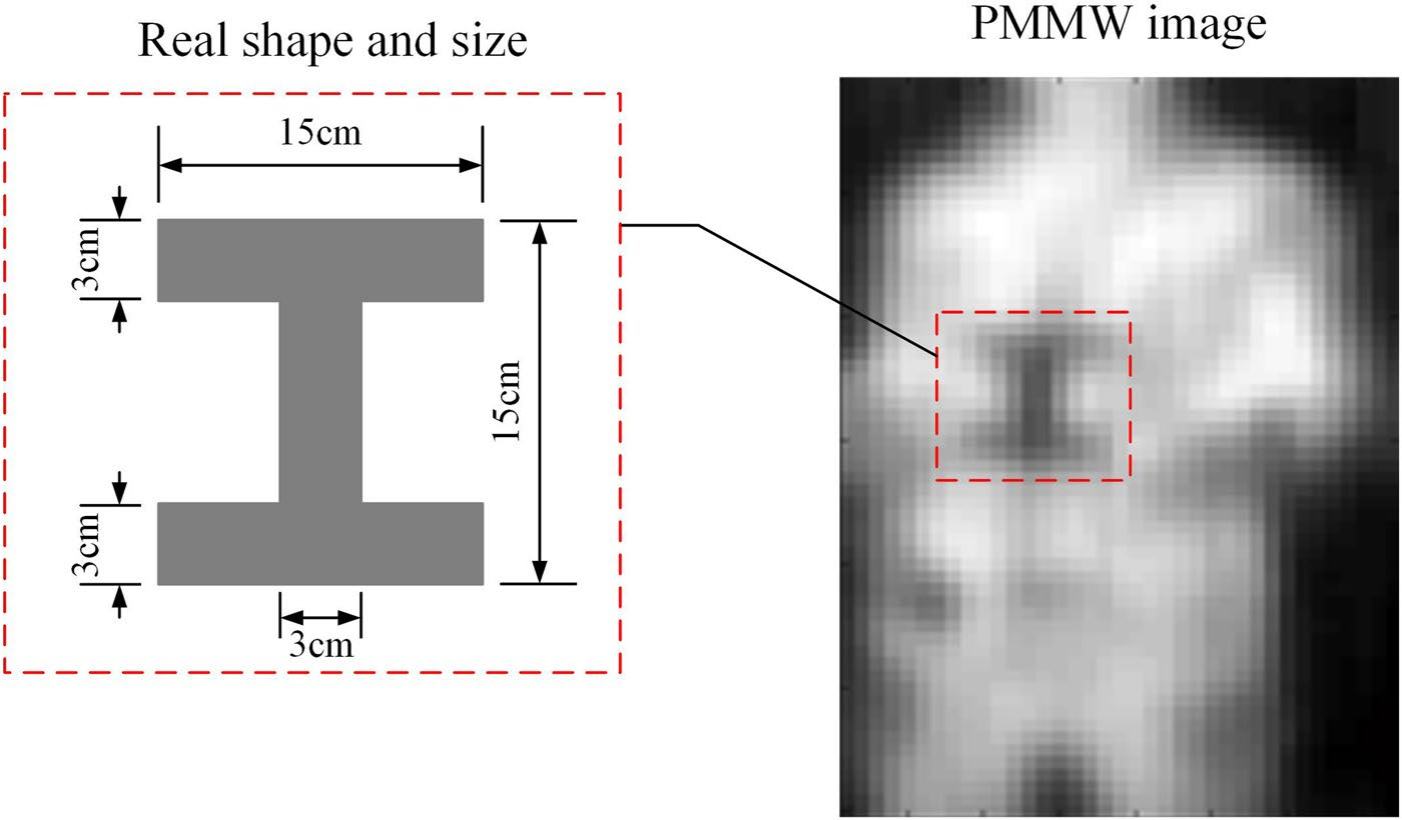
Test of I-shaped metal plate.

To study the resolution of the prototype system quantitatively, an I-shape metal plate as shown in Fig. 11 is fabricated and tested. The width of the narrowest part of the I-shape metal plate is 3 cm. It was held by the same volunteer right in front of his clothes. Experiment was conducted in the same workshop in summer 2016. Even though the test situation is likewise very unfavorable, the I-shape metal plate can be easily identified from the reconstructed Image. From this point of view, the prototype PMMW imaging system is able to detect objects of about 3 cm.

### Metal Toy Gun

**Figure 12:**
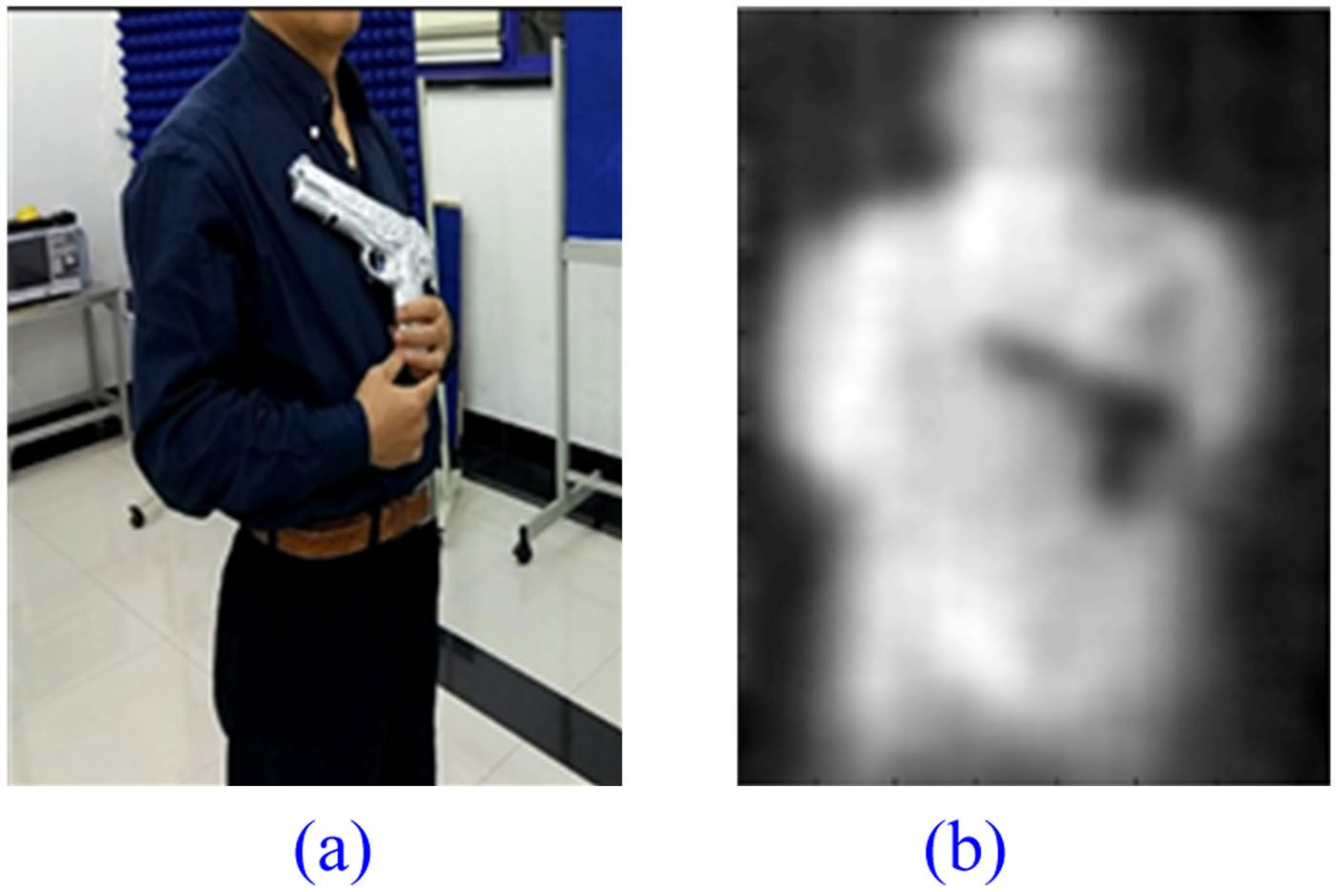
A Volunteer Holding a Metal Toy Gun. (**a**) Photo of Real Scenario, (**b**) Imaging Result.

A photo of a volunteer holding a metal toy gun standing 3 meters away facing the prototype is given in Fig. 12(a). No absorbing wall is used due to more favorable test condition in autumn. The metal toy gun is about 3 cm wide. It is taken out of the dark blue shirt for demonstration purpose. It is hidden in the shirt during actual test. The image displayed on the system terminal is presented in Fig. 12(b).

From Table 1, we know that metal objects are ideal reflectors that do not emit electromagnetic waves by themselves and just reflect the radiation of the surrounding environment, which is lower than that of human body. So the metal toy gun can be clearly detected.

### Metal Knife with Plastic Handle

**Figure 13:**
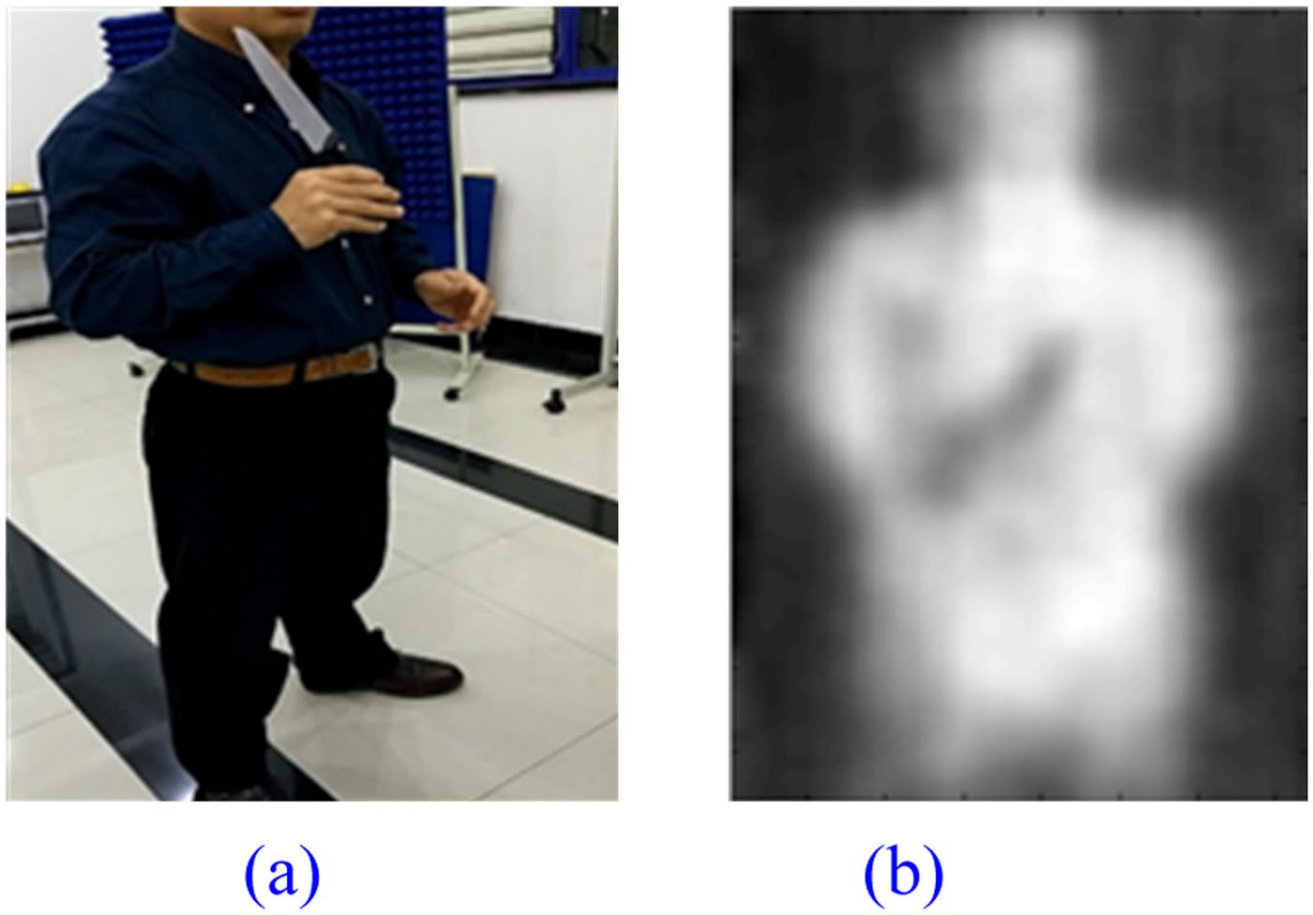
A Volunteer Holding a Metal Knife with Plastic Handle. (**a**) Photo of Real Scenario, (**b**) Imaging Result.

The same volunteer goes through another test holding a metal knife with plastic handle as shown in Fig. 13(a). The blade of the metal knife is about 2.5 cm wide, narrower than even the slender barrel of the metal toy gun. The image displayed on the system terminal is presented in Fig. 13(b). Detection of the metal knife is undoubtedly successful. However, compared with the larger metal toy gun, the image of the detected metal knife is blurrier, especially the part of the plastic handle.

### Plastic Toy Gun

**Figure 14:**
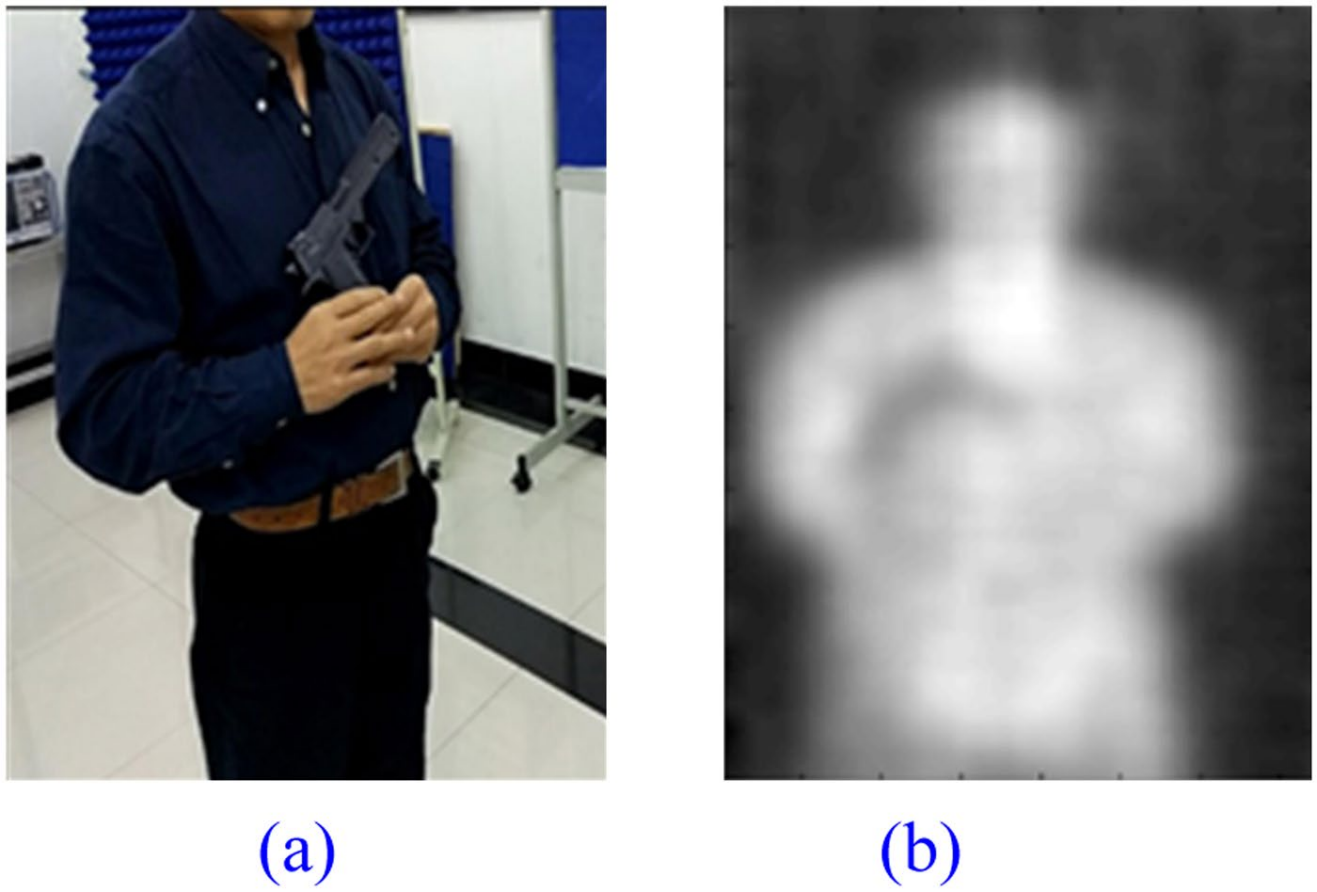
A Volunteer Holding a Plastic Toy Gun. (**a**) Photo of Real Scenario, (**b**) Imaging Result.

One of the disadvantages of wide spread security check system based on metal detection by using eddy current lies with its incapability to detect non-metallic items hidden in clothes. PMMW has emerged as one of the promising alternates to face this challenge. Our PMMW imaging system is tested against the plastic toy gun shown in Fig. 14. Our system is the clear winner against this challenge.

### Ceramic Knife

**Figure 15:**
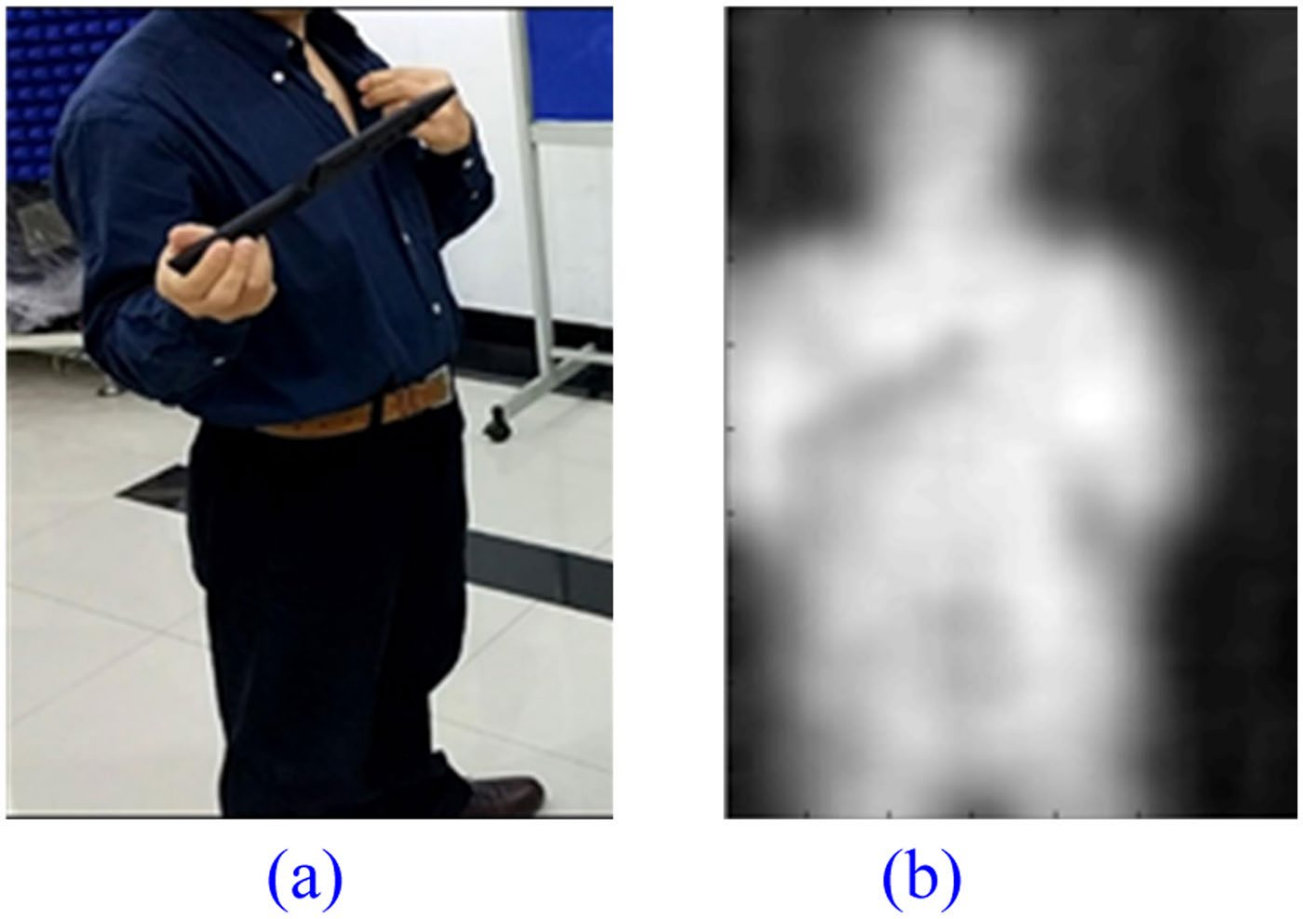
A Volunteer Holding a Ceramic Knife. (**a**) Photo of Real Scenario, (**b**) Imaging Result.

In recent years, ceramic knife has been more and more popular kitchenware. Meanwhile, its threat to security has been more and more imminent. Due to its non-metal characteristics, keeping it out of restricted area such as airport and railway station has been a great challenge. Our novel PMMW imaging systems takes this challenge as shown in Fig. 15. Visually, in terms of size and shape, the tested ceramic knife looks very much like the above metal knife with plastic handle. Undoubtedly, detection of the ceramic knife hidden in clothes is successful.

## Conclusion and Future Work

An efficient, cost-effective, stable and simple passive millimeter wave imaging system is developed in this paper. A more compact and mechanically more stable helical scanning system is invented. Spatial resolution at 3 m is less than 3 cm and the angular resolution is about 0.7°. In addition, the field of view (FOV) is adjustable according to actual target.

Apparently, the preliminary images of indoor imaging results are yet to improve. As discussed in sub section Passive Millimeter Wave Imaging of this paper, the received MMW presents the integrated effect of the tested objects of interest, volunteer’s body, clothes, and the test environment. As such, besides general signal processing approaches to reduce the interference of noise for higher image resolution, with the help of certain a priori information, de-coupling objects of interest from irrelevant surrounding based on electromagnetic theory and statistical analysis might be a more promising solution.

In addition, the prototype imaging system obtains a static image in about 3 seconds. To make it applicable for real time inspection, the efficiency is yet to significantly improve. Several approaches to upgrade the first generation prototype have been under serious scrutiny. Promising approaches and results will be timely presented to the community in follow up publications.
